# 3D printing of micro-nano devices and their applications

**DOI:** 10.1038/s41378-024-00812-3

**Published:** 2025-02-27

**Authors:** Naibo Zhang, Zilai Wang, Zixin Zhao, Dongxing Zhang, Junyu Feng, Linghao Yu, Zhanhong Lin, Qiuquan Guo, Jianming Huang, Junfa Mao, Jun Yang

**Affiliations:** 1 School of Electronic Engineering, Beijing University of Posts and Telecommunications, Beijing, 100876 China; 2 The 54th Research Institute of Electronics Technology Group Corporation (CETC 54), Beijing, 100043 China; 3 School of Shenzhen Institute for Advanced Study, University of Electronic Science and Technology of China, Shenzhen, 518000 China; 4 School of Electronics and Information Technology, Sun Yat-sen University, Guangzhou, 510275 China; 5 Shenzhen University, Shenzhen, 518060 China

**Keywords:** Nanoscale materials, Nanoscale devices

## Abstract

In recent years, the utilization of 3D printing technology in micro and nano device manufacturing has garnered significant attention. Advancements in 3D printing have enabled achieving sub-micron level precision. Unlike conventional micro-machining techniques, 3D printing offers versatility in material selection, such as polymers. 3D printing technology has been gradually applied to the general field of microelectronic devices such as sensors, actuators and flexible electronics due to its adaptability and efficacy in microgeometric design and manufacturing processes. Furthermore, 3D printing technology has also been instrumental in the fabrication of microfluidic devices, both through direct and indirect processes. This paper provides an overview of the evolving landscape of 3D printing technology, delineating the essential materials and processes involved in fabricating microelectronic and microfluidic devices in recent times. Additionally, it synthesizes the diverse applications of these technologies across different domains.

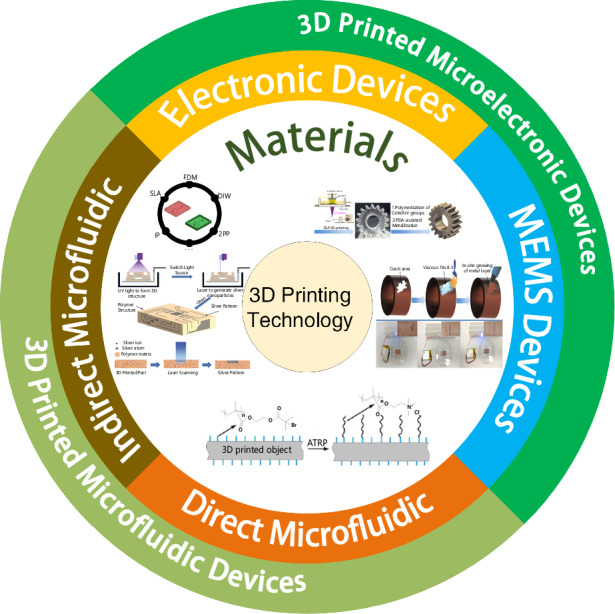

## Introduction

In recent years, the rapid development of micro and nanotechnology has brought unprecedented opportunities and challenges to various fields. Traditional methods for manufacturing micro and nanodevices are limited by complex structures and difficulties in precise size control^1^. 3D printing technology, as an emerging manufacturing method, has gradually gained widespread attention in the field of micro and nanodevices.

3D printing technology achieves device fabrication by layer-by-layer stacking of materials. Compared to traditional micro and nanodevice fabrication methods, 3D printing technology enables the creation of intricate structures with complex internal features, enhancing device functionality. Additionally, 3D printing directly translates digital models into physical structures, simplifying the manufacturing process and allowing for greater customization and precision.

Although 3D printing technology offers flexibility in rapid manufacturing, and strong customization, its applications in micro and nanodevice fabrication are still under development, and their repeatability has not been fully validated^2^, due to the limitation of working materials and fabrication resolution. Furthermore, the lower mechanical performance of 3D-printed parts often requires 3D printing incorporated with other processes for manufacturing^3^.

With the continuous development and improvement of 3D printing technology, an array of innovative materials has emerged for its application. These include ink material, biological materials, composite materials, ceramic materials^4^ and polymer materials^5^. Researchers are increasingly exploring its application in the manufacture of micro and nano devices. For example, in the area of developing biomedicine, 3D printing can produce complex biological structures by biomaterials^6^. In microelectronics, 3D printing technology can be used in the preparation of micro sensors and actuators, as well as in the preparation of microfluidic chips with complex structures^7^. Moreover, in optics, 3D printing technology can be used to manufacture optical components with micro and nano structures for optical communication and photon sensing^8^.

This paper offers a comprehensive overview of the current advancements in 3D printing technology and its diverse applications in micro and nanodevice manufacturing. It thoroughly explores the applications and recent advancements of 3D printing technology in microelectronics and microfluidic devices. By providing a comprehensive synthesis of relevant research, this paper aims to serve as a guiding resource, inspiring further exploration and innovation in this domain. It endeavors to empower researchers to contribute to the continual advancement and practical application of 3D printing technology in micro and nanodevice manufacturing.

## 3D printing technology

The American Society for Testing and Materials (ASTM) has classified 3D printing technologies into seven categories to provide a systematic framework for understanding and categorizing these technologies^9^. They are binder jetting (BJ), directed energy deposition (DED), material extrusion (MEX), material jetting, powder bed fusion (PBF), sheet lamination (SHL), and vat photopolymerization (VP). Each category represents a different approach to 3D printing and has its own unique characteristics and applications, especially in the field of Micro-Electro Mechanical System (MEMS), microelectronics, and microfluidic devices. This section briefly describes the working principles and characteristics of seven additive manufacturing methods.

### Binder jetting

Binder jet (BJ) printing involves liquid binder spraying on powder materials. It uses an inkjet printhead to spray the adhesive into the powder on the powder bed, thereby binding a layer of powder in a selected area, and stacking on the previous printed layers because of the penetration of the adhesive, so as to create a three-dimensional structure.

BJ is compatible with almost any powder materials^10^. It has been used in printing devices of metals, alloys, bio-polymeric materials, and sand devices. BJ has the advantages of lower thermal residual stress, higher productivity, and better powder recyclability. Especially when making structures of large volume, BJ is superior to other powder printing technologies, with faster speeds and less personnel burden^11^.

In the printing process, the slicing defect, powder spreading defect, single-layer printing defect, multi-layer accumulation defect, and depowder defect caused by factors such as the physical properties of the material would have an impact on the accuracy, size, and physical properties of the device^12^.

The issue of depowder is a crucial one among them, with that in mind, different mechanical parts prepared by BJ can accept different minimum feature sizes. For example, the minimum recommended size for solid bumps is 750 µm, the minimum thin wall thickness is 1–3 mm, and the minimum microchannel can reach 250 µm^13^.

### Directed energy deposition

DED has attracted great attention due to its ability to print metal and potentially any metal alloy system, especially functionally graded materials. In this process, raw material in the form of powder or metal silk is delivered to a substrate that simultaneously focuses an energy source such as a laser beam, an electron beam, or a plasma/arc to form a small melt pool and continuously deposit the material layer-by-layer.

DED has a unique advantage in repairing, modifying parts, and being able to deposit large amounts of material quickly. DED is widely used in the repair of damaged high-value devices, during which new materials can be added^14^, as well as in the preparation of large metal parts for rapid delivery, coating on special materials^15^, alloy design and the preparation of multi-material structures^16^.

According to the types of heat sources and feed forms, DED technology can be divided into laser additive manufacturing (LAM-DED), wire and arc additive manufacturing (WAAM), wire and laser additive manufacturing (WLAM), and wire and electron beam additive manufacturing (WEAM)^17^. The feeding technology is further divided into off-axis and coaxial, in which the DED process of the off-axis feeding device is sensitive to the feeding direction and deposition conditions^18^.

In terms of minimum feature size, LAM-DED can achieve ~380 μm, WAAM can reach 1000 μm, WLAM can extend up to five times the linear diameter, and WEAM can be as small as 1600 μm or less^17^.

### Material extrusion

The MEX process involves melting and squeezing a material through a heated nozzle. The printer places the material on the build platform along the process path obtained through the software, the melted filaments then cool and solidify to form solid objects. MEX has the advantage of easy access to equipment, cheap and wide variety of raw materials, including standard plastics, fiber-reinforced polymers, concrete, metals, ceramics, and multi-material production parts^19^.

The typical configuration of MEX printing technology is to extrude the polymer in the form of a filament through a nozzle after heating, and the extruded material is deposited layer-by-layer on a platform to build a 3D device. Commonly known as Fused deposition modeling (FDM), sometimes referred to as Fused filament fabrication (FFF), this method is a MEX technique on a platform that has been widely used in the consumer market^20^.

The most widely used materials in FDM are polymer materials such as acrylonitrile-butadiene-styrene (ABS) and poly(lactic acid) (PLA), whose thermal fluid properties make them easier to prepare and process^21^. Many other composites have also been proposed for FDM, such as polypropylene (PP), polystyrene sulfone, thermoplastic polyurethane^22^. In the processing process, usually two materials are used at the same time, in addition to the construction of the material itself, but also need to support the support structure of the printing process. The nozzle for extruding the polymer can reach a minimum of 200 μm, the minimum diameter of the filament-like material can be drawn up to 170 μm, and the layer thickness can be up to 150 μm^23^.

In addition to FDM, melt extrusion additive manufacturing (MEM) process forms 3D devices by melting and layering a filament-like hot melt material through a liquefier, powder melt extrusion additive manufacturing (P-MEM) process using powder materials. Parts prepared by MEX are widely used in the automotive, electronics, biomedical, construction, aerospace, and home appliances industries^24^.

### Material jetting

Material injection (MJ) technology creates parts by curing droplets of liquid photopolymer ejected by nozzle by UV light. The raw material stored in the closed tank is heated and transported to the nozzle, where a layer is sprayed and deposited on the platform before curing. The platform is lowered one height after each layer is cured, and a new layer is processed. After multiple curing, parts are formed, and other materials are required as support structures in the process. MJ technology is based on inkjet technology, so it is also commonly referred to as—inkjet printing (IP)^25^.

IP can be divided into continuous inkjet and drop-on-demand. According to the working principles of the nozzle^26^, IP can also be divided into piezoelectric inkjet printing, thermal inkjet, electrohydrodynamic jet printing (EHD), needle-based printing, aerosol jet printing (AJP), laser assisted printing, and acoustic printing. Notably, direct ink writing (DIW) is a 3D printing technology that is very similar to IP, except that it allows printing of solid-liquid mixed materials^27^. With the development of high-resolution 3D printing technology, DIW can reach a resolution of 1 µm, determined by the print nozzle diameter. EHD can reach a resolution of 30 nm^28^.

3D printers that apply IP such as PolyJet and MultiJet^29^, enable color and multi-material combination and only needs one cure. It has been used in functional devices, self-powered systems, remote sensors, Internet of Things, biotechnology, medicine, biosensing, and diagnostic applications^30^. Due to the advantages of the simple, rapid preparation, low cost, and high accuracy of the IP process, it is now more used in the field of membrane preparation^31^, and it also has good prospects in the field of micro device processing and preparation.

### Powder bed fusion

PBF is a technology in which powder materials placed on a powder bed are melted by an external energy source to form 3D parts, it can quickly form almost any desired device shape. According to the type of energy and the treatment of powder materials, the PBF process can be divided into Direct laser metal deposition (DLMD), Selective Laser Sintering (SLS), Selective Laser Melting (SLM), Electron Beam Melting (EBM), multi jet fusion (MJF) and Laser micro sintering (LMS).

DLMD process works in an inert environment, coating a powder bed at high temperatures with a layer of metal powder, after which the metal powder is selectively melted and sintered by laser. After one layer is finished, the powder bed is moved down to process the next layer and finally form a 3D metal part. Both DLMD and DED can use laser to process metal parts by fusing or melting powder materials. The difference is that DED requires feeding process for material transportation in the processing process, hence DED is more suitable for the repair of metal parts, custom processing, DLMD is more suitable for the overall processing of 3D metal parts. DLMD is widely used in the manufacture of small metal parts and alloy parts in aerospace and other fields^32^.

SLS and SLM are both laser processing powder material processes, the difference is that SLS melts the powder material and uses the melted binder material to bond into three-dimensional parts, while SLM melts the powder and then cools and solidifies directly into three-dimensional parts. They are widely used in aerospace, electronics, automotive, pharmaceutical, biomedical engineering industries^33^. SLS variant called LMS is developed to reach a resolution of 10–50 µm by using submicron powder materials^34^. SLM offers improvements over SLS in terms of product quality, processing time and manufacturing reliability^35^. However, when machining metal materials, SLM requires a support structure to support the overhanging metal structure.

EBM is a powder bed process utilizing electron beams for selective melting of metal powder. Compared to laser-based methods, EBM reduces costs and processing time.

MJF, utilizing infrared lamps as an energy source, is a powder bed process. It involves depositing a solvent that absorbs infrared radiation energy into the designated area of the powder bed, while a water-based detailing agent is sprayed around the contour to enhance part resolution^36^.

### Sheet lamination

The SHL process superimposes sheets of thin layers of material, which are then bonded together using a bonding process between the layers. SHL commonly used raw materials include paper materials, plastic materials, polymers, metals, ceramics^37^.

According to different layer between the bonding process, the SHL process can be classified into Laminated object manufacturing (LOM), ultrasonic additive manufacturing (UAM), Robotic unit based SHL (R-SHL)^38^. LOM layers are bonded using adhesives between layers, UAM uses ultrasonic bonding technology, R-SHL uses robots for interlayer bonding, which can add heterogeneous materials between layers. The thickness of the plate used is ~260 µm^39^.

Short time and low cost are the characteristics of SHL process, it has a wide range of applications in electronic circuits^40^, optical fiber preparation^41^, and mold manufacturing^42^.

### Vat photopolymerization

VP is a technique for curing a specific region of liquid-photosensitive polymers by irradiation with ultraviolet light. According to the different processing methods, VP can be divided into stereolithography (SLA), digital light processing (DLP), continuous digital light processing (CDLP)^43^, mask stereolithography (MSLA)^44^, two-photon polymerization (2PP)^45^, and Direct laser writing (DLW).

SLA is one of the earliest forms of additive manufacturing. The tank is filled with liquid-photosensitive polymer material, and a lifting table is arranged below the liquid level. Ultraviolet light is irradiated by the laser above, and the material is solidified from line to layer according to the set profile. After the end of one layer of curing, the table is moved down, and then the next layer is cured, and finally the three-dimensional structure is formed. In order to improve the resolution and print the three-dimensional structure with high aspect ratio, micro-stereolithography (µSL) has been proposed on the basis of SLA process^46^.

In the SLA process, the ultraviolet light emitted by the laser source is digitally processed by the DMD and then reflected into the liquid photopolymer, which is the DLP process. MSLA is also a DLP process, which uses a pixel mask image constructed by DMD or Liquid crystal display to digitize the illuminated ultraviolet light and irradiate it into a photosensitive polymer. The DLP process can directly generate a layer of images, so the speed is faster than SLA, but the resolution is more dependent on the accuracy of DMD. After each layer is cured, the DLP process stops ultraviolet light, moves the platform, and re-lays the photosensitive polymer, a process that significantly drags out the processing time. CDLP makes use of the interaction between solid, liquid, and gas at the interface, so that the curing process can be stopped without interruption, the platform moves at a constant rate, and the entire structure can be processed at one curing time.

2PP is an additive manufacturing technology proposed according to the two-photon absorption (TPA) principle^47^. Unlike the limitations of other VP processes on photosensitive polymer materials, two-photon absorption can occur in almost any material, and the resolution of the 2PP process can exceed that of single-photon polymerization. 2PP can create true nanoscale structures with a resolution as low as 0.1 μm^48^, and is currently the most used 3D printing technology for the preparation of micro-devices. At present, it has been applied in microelectronics, MEMS, microfluidic devices, and metamaterials. DLW is a multi-photon polymer printing technology derived from 2PP technology, through which the laser can make the material absorb two or more photons in a local area^49^. Because of its high speed, high precision, and high-resolution, VP is widely used in the preparation of functional devices in various fields.

**Fig. 1:**
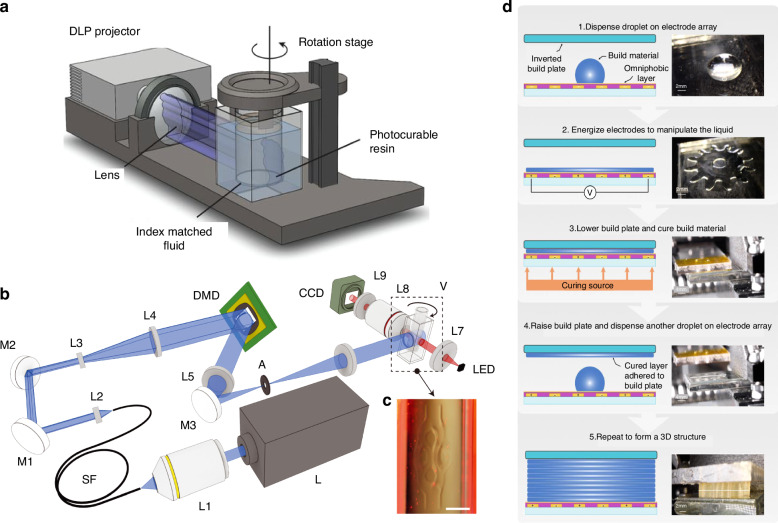
**Schematic of the CAL, the micro-CAL systems, and EFF**. **a** CAL system. DLP projector: digital light processor–based projector^50^. **b** micro-CAL system. L: laser module; L1–L9: lens; M1, M2, M3: mirrors; SF: multimode square core fiber; DMD digital micromirror device; A: diaphragm; V: print container; CCD: Digital Microscope Camera^51^. **c** After light irradiation, the printed object in the container is observed^51^. **d** Electric Field Fabrication printing process^52^. Reprinted with permission from refs. ^50–52^

With the advancement of technology (Table 1), in order to meet the needs of customized preparation, more and more additive manufacturing techniques have been proposed, some are improvements to the above methods, and some are new methods. For example, in view of the difficulty in realizing three-dimensional complexity of additive manufacturing methods such as 2PP compared with traditional micromanufacturing processes, Taylor et al. proposed a computed axial lithography (CAL) method^50^, which can print the whole three-dimensional body concurrently instead of layering, and the speed is several orders of magnitude faster than the layer-by-layer method. This method was achieved by using light to propagate the image projection through the material from different angles. Several years later, they developed a microscale computed axial lithography (micro-CAL) method^51^, which decreased the minimum feature size from 300 µm to 50 µm compared to CAL. The schematic of the CAL and the micro-CAL systems is shown in Fig. 1a–c. Compared to CAL, micro-CAL technology has a more complex imaging system. In addition to printing polymer materials, this method can also prepare fine glass structures. To overcome the challenge of integrating multi-material components during the printing process, Duncan et al.^52^ used liquid dielectrophoresis, or the polarization of dielectric liquids via non-uniform electric fields, to manipulate and shape the entire layer at once, mobilize the droplet liquid into the desired shape for that layer, cure, and stack it on an inverted build plate until the 3D structure is complete (Fig. 1d). This method, Electric Field Fabrication (EFF), had initially manufactured microfluidic channels and films. For the fabrication of MEMS devices, Bickel et al.^53^ combined sputtering, aerosol generation, and plasma injection techniques using a miniature DC/pulsed plasma source. The method named Atmospheric Pressure Sputtering Layer Deposition is a promising technology because it can simplify the manufacturing process and reduce manufacturing costs. There are some other additive manufacturing techniques that are detailed in the next section.

**Table 1:** The properties of seven 3D printing technologies

Technology	Variant	Resolution	Advantages	Application
Binder jetting	-Sand binder jetting -Metal binder jetting	~250 µm^13^	-Lower thermal residual stress -Higher productivity -Better powder recyclability -faster speeds -less personnel burden^11^	-The preparation of metals, alloys, bio-polymeric materials, sand, and other devices
Directed energy deposition	-LAM -DED -WAAM -WLAM -WEAM^17^	~380 μm^17^	-Unique advantages in repairing, modifying parts -Quickly depositing large amounts of material	-The repair of damaged high-value devices^14^ - The preparation of large metal parts for rapid delivery -Coating on special materials^15^ -Alloy design -The preparation of multimaterial structures^16^.
Material extrusion	-FDM -FFF^20^ -MEM -P-MEM	~150 μm^23^	-Easy access to equipment -Low cost -Wide variety of raw materials^19^	-Automotive -Electronics -Biomedical -Construction -Aerospace -Home appliances^24^
Material jetting	-CIJ -DOD -PIP -TIJ -EHD -AJP^26^ -DIW^27^	>0.3 μm^28^	-Enable color, multi-material combination -Simple, rapid preparation -Low cost -High accuracy	-Functional devices -Self-powered systems -Remote sensors -Internet of Things -Biosensing diagnostic^30^ -Membrane preparation^31^
Powder bed fusion	-DLMD^32^ -SLS -SLM -EBM -LMS -MJF^35^	>10 μm^34^	-Form almost any desired device shape quickly	-Aerospace^32^ -Electronics -Automotive -Pharmaceutical -Biomedical engineering
Sheet lamination	-LOM -UAM -R-SHL^38^	~260 μm^39^	-High speed -Low cost	-Electronic circuits^40^ -Glass fiber -Optical fiber preparation^41^ -Mold manufacturing^42^
Vat photopolymerization	-SLA -DLP -CDLP^43^ -MSLA^44^ -2PP^45^ -DLW^49^ **-**µSL^46^	>0.1 μm^48^	-High speed -High precision^48^	-Microelectronics -Microfluidic devices -Metamaterials -Preparation of functional devices

## 3D-printed devices

### 3D-printed microelectronic devices

In recent decades, microelectronic devices had become essential in modern technology, because to their small size, excellent performance, and affordability. The advancement of microelectronic technology has led to the gradual integration of 3D printing methods into various microelectronic devices, including sensors^54^, actuator^55^, and flexible electronics^56^. In the fabrication of electronic devices, 3D manufacturing patterns are frequently evident, various 2D printing methods, including offset, screen printing, and conventional subtractive processes, are employed to create 3D electronic devices. In contrast to the material waste associated with traditional subtractive manufacturing and the requirement for printing templates in 2D printing, 3D printing technology has garnered greater interest from academics due to its capability to produce high-performance, intricately designed electronic devices with limited output^57^.

The flexible printing capabilities and precise printing of 3D printing can be extensively utilized in the fabrication of sensors. The typical approach involves preparing the assisted structure and support structure of the sensor. Wei et al.^58^ employed a self-developed FDM platform to manufacture bionic structures that conform to the shape of the human body. These structures were utilized as supplementary components in multi-directional touch sensors, which may be directly printed into the surface of the human body. Pagliano et al.^59^ employed the 2PP technique to fabricate a support structure for a micro-accelerometer in the form of a cantilever beam. They subsequently conducted metal evaporation deposition to create the functional structure, thus validating the applicability of 3D printing for intricate geometric support structures in micro-nano devices. Bartosz Kawa and his colleagues^60^ utilized IP to fabricate the spring and packaging structure of the vibration electromagnetic energy harvester. Preparing microstructures with high aspect ratio and high complexity is a difficult task when working with soft polymer materials like polydimethylsiloxane (PDMS). In order to address this issue, molds can be rendered through the utilization of 3D printing technology. Kamat and colleagues^61^ devised a technique for utilizing 3D printing to create sacrificial metal tools, which are then used to indirectly fabricate PDMS frame structures with microchannels of 150 micrometers in diameter. This approach enhances the convenience of fabricating PDMS structures with high aspect ratio and more complexity.

Certain sensors include specialized configurations of electrodes, dielectric layers, and thin films, which are crucial for the implementation of sensor functionalities. Due to the ability of certain 3D printing methods to achieve submicron resolution and create high aspect ratio structures, it has becoming extensively utilized in the fabrication of specialized sensor devices. Yuk et al.^62^ constructed electrode arrays with excellent resolution and high aspect ratio for brain probes. Their method of transforming low-viscosity fluids into physical gels resolves the limitation of 3D printing in handling highly fluid conductive polymers. Taylor et al.^63^ employed the EHD approach to fabricate thin films with an average thickness of less than 100 nm as transduction components for a gas sensor. Zhao et al.^64^ utilized PDMS materials to fabricate a distinctive dielectric layer in the form of a frog’s leg as a component of a capacitive wearable flexible sensor, thereby reducing the time and expenses associated with sensor production.

Furthermore, the sensor can also be utilized in the process of preparing functional component. Functional polymer materials and composites have advanced the application of 3D printing in the creation of functional components. For instance, piezoelectric polymers that are appropriate for use in piezoelectric sensors^65^. The properties of these materials undergo changes over time due to external influences, so bringing the element of time to 3D printing, also referred to as 4D printing.

MEMS actuators commonly utilize 3D printing to fabricate intricate structures, support structures, and soft robots^66^, it always combined with micromachining processes. Hashimoto et al.^67^ employed the 2PP method to fabricate the vertical spiral structure of the electrothermal actuator, serving as the construction framework for the double-layer electrothermal actuator. Subsequently, they achieved electrothermal control by introducing liquid metal into the spiral structure. Simultaneously, certain MEMS actuators are entirely fabricated using 3D printing technology. Lee et al.^68^ fabricated a dual-port MEMS switch with the FDM technique, employing conductive PLA as the electrode material and polyvinyl glycol (PVA) as the sacrificial layer. While the current ratio between the two states increased, the switching gap expanded to 160 µm, surpassing the size of typical MEMS switches. An excessively wide gap will lead to an inability to effectively integrate into the communication system for application purposes. By utilizing additive manufacturing principles, 3D printing allows for the direct production of MEMS devices layer-by-layer, eliminating the need for traditional fabrication processes and enabling the integration of intricate designs and functionalities.

The cost of a single device in the conventional micromachined MEMS device manufacture method typically decreases as the number of devices needed increases^69^. To achieve cost reduction, there is a greater emphasis on the production of high-volume MEMS processing, while disregarding the requirements of small-scale markets or devices used for experimental purposes. The integration of 3D printing technology in the preparation process of MEMS devices can significantly decrease expenses.3D printing offers cost-effective advantages for producing MEMS devices in small quantities. Its flexible printing capabilities and high precision make it suitable for creating high aspect ratio structures, complex construction structures, and assisted structures for MEMS sensors and actuators. Functional polymer materials and ink materials have reached a level of maturity that allows them to be utilized in the production of functional components. Furthermore, this approach facilitates the incorporation of numerous components or capabilities into a solitary printed device, hence minimizing the need for assembly and enhancing the overall performance of the device. 3D printing allows for quick creation of prototypes and iterative design procedures, making it easier to build and improve MEMS devices. The 3D printing technology provides significant flexibility and feasibility in terms of micro geometric design and fabrication compared to subtractive manufacturing methods like machining and laser cutting. This process allows for precise control over the design, materials, and performance of micrometer-sized devices^70^.

### Materials for 3D-printed microelectronic devices

Extensive and thorough research has been conducted on the development of new materials for 3D printing. Notably, there have been significant advancements in polymer materials and ink materials, with many of them demonstrating conductivity and functionality. Examples include conductive inks^66^, conductive polymers^62^, and piezoelectric composites^71^. These sophisticated materials possess exceptional mechanical strength, outstanding electrical conductivity, and biocompatibility. They are already extensively utilized in the field of 3D-printed electronic devices. Selecting the appropriate material^72^ can enhance the efficiency of microelectronic devices and save production expenses.

Conductive ink such as MXenes has been widely used in microelectronic products such as flexible sensors and miniature energy storage devices^73^ due to their high metal-like conductivity and excellent electrochemical properties. Researchers have adapted the synthesis route of MXenes nanocomposites for applications in microelectronic devices. Especially in electrochemical energy storage devices represented by micro-supercapacitors (MSCs), which can be directly coupled to microelectronics as an independent microscale power source, overcoming the inherent shortcomings of traditional electrochemical energy storage devices^74^. And MXenes are considered to be an unbeatable choice on high-performance electrode materials for MSCs. For example, Sun et al.^75^ constructed IP ink based on metal-conducting N-MXene and NiS highly electrochemically active heterostructures by loading porous NiS onto N-MXene materials. It was used for high-precision fabrication of planar asymmetrical micro-supercapacitors (AMSCs) with excellent volumetric capacitance and outstanding integration capability Compared with conventional MSCs (0.6 V), this ink produces high-performance AMSCs with a larger voltage window (1.5 V) and better integration capability. In addition to high electrical conductivity, MSC mechanical properties are also optimized for further development of MXene composite inks. For example, Li et al.^76^ prepared a temperature-resistant, electrically conductive, self-repairing, and highly adhesive nanocomposite hydrogel by doping MXene into a polymer network consisting of a water-glycerol binary solvent system. The mechanical properties (e.g., tensile elongation ~1000%) and electrical conductivity (~1.34 S/m) were significantly enhanced with the incorporation of low-content (0.1–0.3 wt%) MXene flakes with high sensitivity (GF = 3.93) and wide detection range (up to 600% in cyclic testing). This multifunctional MXene nanocomposite hydrogel may bring new outlook and potential for the design and development of flexible sensors for complex environmental applications. Furthermore, conductive inks can be created by the process of particle doping and the combination of many materials. Zhang et al.^66^ synthesized an ion conducting elastomer (ICE) by combining poly(acrylic acid) (PAA) with ionic monomers, namely ammonium propane sulfonate (DMAPS), and an ionic liquid. To enhance its mechanical properties, silica nanoparticles were included into the elastomer. This ink substance is suitable for utilization in DIW technology. The material has a significant level of mechanical flexibility, with a uniaxial tensile rate of 720%. Its electrical characteristics remain unaffected by both tensile and cyclic deformation, indicating strong resilience. Binelli et al.^77^ incorporated carbon black particles into surface-modified cellulose nanocrystals to create a porous network with customizable rheological characteristics and stiffness. This allowed for the alteration of electrical resistance in response to external pressures. Hou et al.^78^ synthesized conductive inks by including graphene nanoplates and carbon nanotubes (CNTs), which can be employed as printing materials for MJF technology. The sensors produced with this material not only boosted the mechanical and flame retardant characteristics, but also improved their stability and sensitivity. Introducing metal particles into the ink will confer it with conductive characteristics. Kim et al.^79^ have fabricated LC circuits by utilizing DIW approach with conductive silver ink. This ink substance is inexpensive and simple to create.

**Fig. 2:**
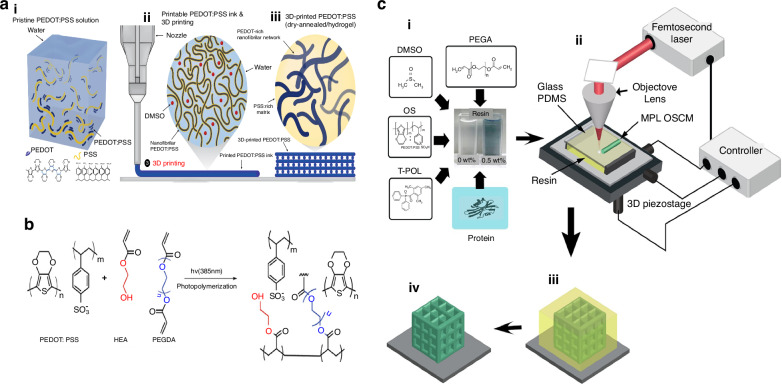
**3D printing material and preparation method**. **a** (i) Pristine PEDOT: PSS. (ii) Pristine PEDOT: PSS solution can be converted into a 3D printable conducting polymer ink by lyophilization in cryogenic condition and re-dispersion with a solvent. (iii) 3D-printed conducting polymers can be converted into a pure PEDOT: PSS both in dry and hydrogel states by dry-annealing and subsequent swelling in wet environment, respectively^62^. **b** Photopolymerization scheme of PEDOT: PSS ink. PEGDA and HEA react throughout a radical polymerization initiated by the photoinitiators, creating an interpenetrated network with PEDOT: PSS^85^. **c** Resin components and MPL fabrication process. (i) Components of the OS composite resin. (ii) Experimental setup for the MPL process. (iii) The OS composite resin (yellow color) is crosslinked by a focused fs laser to create 3D OSCMs (green color). (iv) The sample is then rinsed in ethanol to remove any unsolidified resin, leaving the 3D OSCMs on the substrate^86^. Reprinted with permission from refs. ^62,85,86^

Microelectronic devices with high power densities and fast charge/discharge rates are well suited as power sources for implantable bioelectronic devices, however, this requires materials with excellent biocompatibility and durability. Advancements in materials are necessary to bridge the gap between biology and electronics, resulting in improved device performance or the development of completely new device concepts^80^. Notably polymer composites have the required biological properties. They are frequently utilized in the conducting interlayers^81^ and electrodes^82,83^ of bioelectronic devices. In terms of combining with 3D printing technology, Krishnadoss et al.^84^ proposed the synthesis of BIL-functionalized hydrogel electrolytes by reacting choline acrylate with the biocompatible polymer GelMA. The ability of the electrolyte to penetrate the porous high-surface electrodes was experimentally shown to enable these MSC devices to have high capacitance retention and high power and energy densities. This electrolyte material platform has robust mechanical properties and biocompatibility, enabling direct printing of complex structures for 3D bioelectronic devices. Various materials, including organic semiconductor materials and graphene oxide (GO), are used in the construction of microelectronic devices and microstructures via 3D printing technology. A conductive polymer ink based on poly(3,4-ethylenedioxythiophene): polystyrene sulfonate (PEDOT: PSS) was developed (Fig. 2a)^62^. To fabricate various microelectronic devices such as flexible circuits and soft nerve probes. A high-resolution conductive ink was developed by PEDOT: PSS formulated in a photocurable matrix consisting of polyethylene glycol diacrylate (PEGDA) as a crosslinker and a mixture of riboflavin/Irgacure 2959 as a photoinitiator (Fig. 2b)^85^. Different copolymers were prepared with PEGDA to reduce crosslinking degree and improve mechanical properties. The viscoelastic behavior of the hydrogel network after ultraviolet irradiation was tracked by photo rheology technology. After UV exposition for 5 s, the gel reaches a plateau with storage and loss moduli of 1 × 106 Pa, demonstrating the ability of the ink to reach the gel point before achieving complete transformation. This photopolymerization can obtain 3D structures very quickly and is the best choice for 3D printing. Dadras-Toussi et al.^86^ introduced a uniform and see-through resin that is sensitive to light and contains organic semiconductor materials (OS) (Fig. 2c). They used a process called multiphoton lithography (MPL) to create 3D microstructures with excellent structural characteristics. This method was employed to produce a range of 3D composite microstructures (OSCMs) and microelectronic devices. Adding 0.5 wt% OS to the photosensitive resin can significantly boost the electrical conductivity of the composite polymers by around 10 orders of magnitude. In contrast to composite resins that contain carbon-based nanomaterials like CNTs and graphene, this OS composite polymer (with 0.5 wt% OS) can significantly increase electrical conductivity by around 10 orders of magnitude. A material containing 5 weight percent of OS (oxidized silicon) exhibits both a high specific conductivity (about 5.4 × 10^4^ Sm^−1^ wt%^−1^) and a flat surface, making it suitable for producing superior 3D microstructures. They incorporated laminin (LN) into OSCM^87^, which significantly enhanced the adhesion of biological cells to the 3D-printed microstructures. This characteristic has significant potential for many biological applications, including flexible microelectronics and wearable biosensors. Furthermore, aqueous suspension of GO nanoflakes^63^ is utilized for a cost-efficient film deposition method that eliminates the need for any subsequent processing. Polymers such as PDMS^61^, PVA^68^, PLA^88^, and photocurable resin^89^ are also commonly used to manufacture support structures and auxiliary structures in microelectronic devices. Zhou et al.^90^ utilized DLP printing to create an auxetic structure using aliphatic urethane diacrylate. They harnessed the synclastic effect of this structure to capture bending energy using the 3–1 mode piezoelectric effect. The application constraints of piezoelectric nanogenerators for flexible polymer sheets are surpassed.

Soft conductor materials are considered to be revolutionary materials for future applications such as electronic skin and soft robotics due to their conductive, soft and stretchable properties. However, soft conductor materials are susceptible to wide range of temperature variations due to the incompatibility of their conductive domains with the elastic network in the development of electronically conductive to ionically conductive materials. Based on this problem, Lei et al. proposed a stretchable conductor, which is provided with charge carriers by small molecule-like liquid electrolytes (e.g., ILs), and polymers with a similar structure for synergistic interaction, thus ensuring the structural stability. The researchers printed the stretchable conductor directly onto a dielectric elastomer (VHB) with a three-layer structure. The researchers used the conductor to fabricate a transparent and integrated system that can be used to mimic the mechanoreceptors, humidity receptors, and thermoreceptors of natural skin. Lv et al. developed a stretchable conductor with excellent electrical properties by synthesizing a sustainable and recyclable vegetable oil polyurethane (VegPU) elastomeric binder and developing a solution sintering method for composites with silver flakes^91^.

**Fig. 3:**
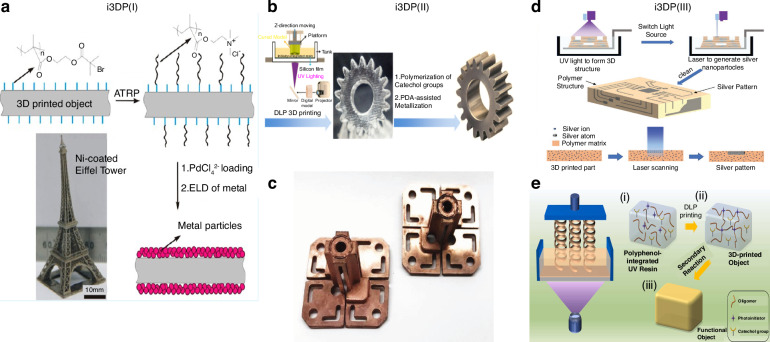
**3D-printed electronics achieved through the integration of active initiators into printing materials, known as i3DP (initiator-integrated 3D printing)**. **a** incorporating a vinyl-terminated initiator into UV-curable resin, referred to as i3DP (I)^93^. **b** i3DP (II) involves the integration of a bioinspired initiator, dopamine^96^. **c** antennas with polarized dipoles created using i3DP (II)^98^. **d**–**e** i3DP (III) integrates AgNO3 and polyphenols for direct surface modification^97,100^. Reprinted with permission from refs. ^93,96–98,100^

The utilization of unique materials in 3D printing for the production of functional components is receiving growing interest. Integrating active initiators into printing materials has emerged as a highly promising avenue for advancing the field of 3D printing microelectronics. Yang et al.^92^ pioneered the development of i3DP (initiator-integrated 3D printing) technology by initially incorporating a vinyl-terminated initiator into UV-curable resin (Fig. 3a). This innovation resulted in the creation of functional structural materials capable of facilitating post-printing surface-initiated modification. Leveraging the capabilities of 3D printing and surface-initiated atom transfer radical polymerization, i3DP offers a viable solution for achieving complex 3D-printed architectures with tailored surface modifications to suit diverse applications. Examples include ultralight and fully recoverable deformable electrodes^93^, magnetically manipulated robots^94^, and electromagnetic shielding electronics^95^.

Continuing along this trajectory, the Yang research group subsequently developed the second generation of I3DP, referred to as i3DP (II)^96^, followed by the third generation, known as i3DP (III)^97^, as (Fig. 3b,d). In i3DP (II), a bioinspired initiator, dopamine, was introduced into photocurable polymers, resulting in electronics with enhanced electrical properties, such as polarized antennas^98^, as (Fig. 3c). Notably, this approach enables in situ repair of damaged conductive structures, thereby extending product lifespan and addressing the persistent challenge of repairing 3D-printed parts^96^. In efforts to streamline the fabrication process, initiators capable of direct surface modification were proposed, including AgNO3 and polyphenols. AgNO3 can be transformed into conductive Ag particles through laser irradiation, while polyphenols enable direct surface modification of 3D-printed objects (Fig. 3d, e). This methodology has been successfully applied to the creation of conformal electronics, flexible electronics, and volumetric electronics^97,99,100^.

Piezoelectric materials have garnered significant interest owing to their distinct characteristics. 3D printing technology provides a novel method for producing piezoelectric materials with intricate geometric forms, which may be challenging or expensive to accomplish using conventional manufacturing methods. The ceramic-polymer composites BaTiO3-polyvinylidene fluoride (PVDF) and BaTiO3-PEGDA with piezoelectric properties can currently be fabricated using 3D printing technology^71^. Bodkhe et al.^101^ conducted a study where they compared three methods: ball milling, extrusion mixing, and sonication. The purpose was to add varying concentrations of BaTiO3 nanoparticles to PVDF and examine their ability to initiate and maintain the β-phase in PVDF. Additionally, they aimed to develop a nanocomposite formulation that would achieve a balance between printability and piezoelectric properties similar to commercially available PVDF films without the need for poling. This material is suitable for 3D printing with the assistance of solvent evaporation. Currently, the majority of 3D-printed piezoelectric devices are predominantly produced using PVDF or BaTiO3-PVDF^65^. The future direction of study is in the functionalization of PVDF and the exploration of different piezoelectric polymers and ceramics.

#### Application of 3D-printed microelectronic devices

The high-performance mechanical flexibility and excellent stability of 3D-printed microelectronic devices largely meet the demand for flexible microelectronic devices brought about by the rapid development of smart wearable and biomedical sensors at this stage. Current research is mainly focused on the scalable fabrication of microelectronic devices, such as flexible sensors^102^, e-skin^103^, energy storage devices^104^, etc.

Flexible microelectronic devices are able to maintain their shape stability and their functional properties in the presence of mechanical bending and stretching. To meet such requirements, polymers^105^, Conductive inks^28^, and stretchable conductor^106^ are already widely used in today’s 3D-printed microelectronic devices.

**Fig. 4:**
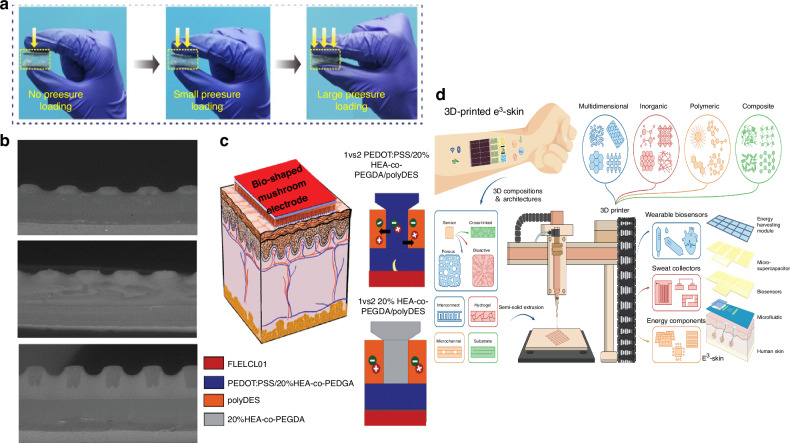
**Application of 3D printing wearable devices**. **a** Optical images of the frog leg structural sensor under different pressure-loading states^64^. **b** SEM images of the conducting mushroom with the DEM^85^. **c** Left: Scheme of the mushroom-shaped electrode adhered on the skin for epidermal bio potential detections of electromyography (EMG), Right: Schematic representation of the different multi-material printed electrodes^85^. **d** Schematic illustration of the SSE-based 3D printing^110^. Reprinted with permission from refs. ^64,85,110^

Wearable sensors can monitor and analyze in real time the physical, biological and chemical information provided by our skin. Strain sensors, for example, are used as devices that can convert mechanical deformation signals into electrical signals, and have become one of the core of devices in the gradually developing field of intelligent detection. For example, a flexible capacitive pressure sensor are proposed^64^ utilizing a three-dimensional (3D) array of bionic frog leg structural composites as a dielectric layer (Fig. 4a). The bionic composite layer can be shortened at the upper and lower poles thereby improving the dielectric properties. The researchers evaluated the sensor with a wide pressure detection range (0–200 kPa), ultra-low detection limit (0.5 Pa), ultra-high sensitivity (0.583 kPa, 0–1.2 kPa), fast response/recovery characteristics (40 and 45 ms, 1 kPa), and long-term stability, as assessed by a stress test. Dominguez-Alfaro et al.^85^ fabricated a conductive mushroom electrode with PEDOT: PSS/20%HEA-co-PEGDA combined with deep eutectic monomers (DEM) (Fig. 4b). The electrode has adhesion and mixed ion-electron conductivity, and is used to attach to the skin as the biopotential signal recording of muscle groups (Fig. 4c). Lin et al.^107^ employed FDM to fabricate three distinct thermoplastic elastomer dielectric layer structures for capacitive force sensors. They examined how the structural design affected sensor sensitivity and confirmed that 3D printing technology can be used for future sensor manufacturing. A fully 3D-printed flexible haptic sensor based on bionic interlock and negative Poisson ratio structure for sensing contact pressure and ambient temperature^58^ are manufactured. The sensor is made of CNT/graphene/silicone composite. silver-coated copper/silicone composite is used as the printing material for the electrode. When external pressure is applied, the papilla bionic structure deforms, the conductivity of the composite material changes, and the pressure signal is formed by electrode detection. Qiu et al. incorporated SiO_2_ particles into the liquid metal to improve its poor rheology and wettability. They used commercial 3D printing equipment to create a liquid metal sensing system based on this composite material, integrated into the glove, and with the help of deep learning algorithms, can realize the recognition of boxing technology^108^. A digital platform based on DIW was developed for integrated manufacturing of silicon-based soft wearables with embedded piezoresistive sensors. A smart insole was fabricated by this platform to measure gait force in the field during physical activity^77^. Insoles can be utilized for pressure sensing^79^ by employing DIW to manufacture LC sensors and FFF to manufacture the insoles. These insoles are then combined with unipolar antennas, allowing them to function as wireless pressure sensors. Brown et al.^109^ introduced a technique for producing electrode arrays using DLW technology. This method is compatible with both standard silicon and flexible polyimide device manufacturing processes. It enables the production of electrodes in various distinctive shapes, offering a novel tool for neuroscience research. These studies provide new ideas for future applications of this next generation of flexible electronics.

E-skin is an artificial skin that replicates the sensory capabilities of human skin. It incorporates many sensors to continuously monitor real-time information about the human body. Song et al. have developed a 3D printing process named semisolid extrusion (SSE) based on DIW technology and a phase elimination strategy for selective removal of sacrificial components in inks (Fig. 4d). They created an epifluidic elastic electronic skin (e^3^-skin) that can monitor glucose, alcohol, and pH levels in sweat in real time, as well as heart rate and temperature, which, combined with machine learning methods, can enable multimodal health detection^110^.

MSCs with excellent electrical properties and flexibility are widely used as power supply schemes in wearable electronic devices. Tang et al.^111^ presented low-cost 3D printing of MSCs based on Fe_2_O_3_/graphene/Ag electrodes, and experimentally demonstrated that the capacitance retention was as high as 89% over thousands of charging and discharging cycles. the MSCs also exhibited excellent device flexibility during bending cycles. Chae et al.^112^ presented a method for fully 3D-printed solid-state energy storage devices, in which 3D-printed fluids for the collector, electrodes, and electrolyte layers were sequentially printed. The MSC devices prepared by this method operated stably at voltages up to 3 V with energy densities ranging from 25.4 to 8.5 μWh-cm and power densities ranging from 150 to 6483 μW-cm, respectively.

In electronic circuit equipment, 3D printing technology offers an innovative approach to three-dimensional manufacturing, enabling the simultaneous printing of the entire circuit—including substrate, conductive pathways, and passive components—through multi-material printing, in contrast to conventional printed electronics processes. The multi-material printing method offers benefits such as securing local supply, minimizing material waste, decreasing environmental pollution, lowering energy consumption, and shortening production cycles, thereby attracting the interest of researchers. The multi-material printing system can utilize FFF, DIW, AJP, IP, PBF, and VP technologies independently, or function as a hybrid system including various 3D printing technologies^113^. Nano Dimension’s Dragonfly IV^114^ is a multi-material printing system utilizing IP technology, capable of simultaneously applying conductive and dielectric inks to produce dielectric layers and conductive lines; however, it lacks the capability for automatic component embedding and requires the printing process to be manually concluded. Aiming at the component embedding method of multi-material printing, Ghazali et al.^115^ proposed a component embedding technique for multi-material printing that integrates 3D printing with metal sputtering, applied to various RF devices such as antennas and filters, serving as an enhancement to the multi-material printing system. The development of 3D printing for passive components, including resistors, capacitors, and inductors, has occurred. Flowers et al.^116^ employed FFF technology to fabricate passive devices utilizing dual-material fuses, showcasing the capability to embed and interconnect surface-mounted components within 3D-printed structures. They demonstrated the capability of 3D printing to fabricate intricate three-dimensional circuits consisting of embedded or entirely printed electrical components. Wittkopf et al.^117^ proposed a holistic approach to manufacturing printed circuits using MJF and AJP. The limited shelf life of inks is a prevalent disadvantage of multi-material printing systems, Vorunichev^118^ assessed the printing efficacy of expired nano-inks and discovered that utilizing ink that had expired a year prior did not compromise the quality and resolution of the printed circuit board; however, it necessitated frequent filter replacements to avert the formation of clots in the ink.

The integration of 3D printing technology with MEMS has opened up new horizons for sensor fabrication and design.

MEMS Energy harvester is the common way to power low-power sensor devices, and MEMS energy harvesters have been booming in recent years^119^. The first use of 3D printing in the energy harvester is to use FDM to prepare the shell of it^120^. After that, FDM and SLA^121^ processes were used to prepare the active mechanical components of the energy harvester, but due to structural limitations, the volume of the prepared energy harvester was greater than 1 cm^3^ in most cases. An electromagnetic energy harvester using IP technology, combining the 3D-printed micromechanical structure with micro permanent magnets and microelectronic coils was proposed, which can generate 24 μW of power with an overall volume of less than 1 cm^3^, which is the smallest 3D-printed energy harvester reported so far^60^. DLP was used to prepare piezoelectric nanogenerators based on polymer films^90^,the device uses synclastic effect for energy harvesting for the first time and is used as a flexible energy harvesting device.

3D printing is also used in the preparation of MEMS physical sensors. 3D printing enables the incorporation of novel materials and composites into sensor designs. Traditional sensors are typically limited to specific materials due to the constraints of the manufacturing processes. However, 3D printing can accommodate a wider range of materials, including conductive polymers, nanomaterials, and biocompatible substances, enabling the development of sensors with enhanced properties and tailored functionalities. This versatility in material selection opens up possibilities for the creation of sensors optimized for specific applications.

Blankenship et al. have developed a 3D printing method based on 2PP technology that constructs quantum sensing particles (nitrogen-vacancy centers) into 3D structures capable of accurately detecting temperature and magnetic field changes in microscopic environments^122^. Ye et al.^88^ applied the FDM process to prepare a low-cost, small-size, long-life mechanical wind sensor using a commercially available 3D printer. They printed the crossed cantilever beam, cylindrical sensing structure located in the center and four strain gauges of the sensor with PLA.

3D-printed high aspect ratio microchannels provide a new solution for gas or liquid sensors. Hawke et al. printed a microfluidic tube, which was part of a compact, low-cost, and responsive flow sensor. The flow was measured through the viscous pressure drop between the two sensors^123^. Ollé et al.^124^ developed a microfluidic gas detector using 3D-printed polymer microchannels to monitor volatile organic compounds within a vehicle.

**Fig. 5:**
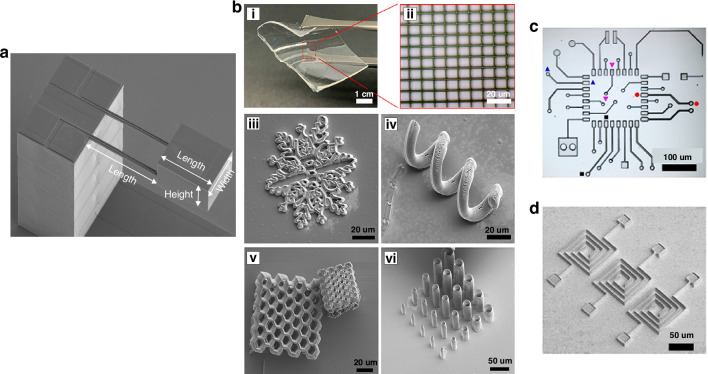
**3D printing micro-nano devices**. **a** SEM image of the 3D-printed accelerometer structure^59^. **b** 3D microfabrication of conductive and bioactive microstructures based on OS composite resin via MPL and resin transparency. (i) Thin and flexible PDMS substrate used for MPL fabrication. (ii) Optical microscopy micrograph of a micro-grid fabricated on PDMS film shown in (i). (iii) Micro-snowflake. (iv) Micro-spring. (v) Micro-honeycomb (vi) Vertical micro-tube^86^. **c** Optical microscopy micrograph of a μPCB comprised of various electrical elements^86^. **d** SEM micrograph of a microcapacitor array^86^. Reprinted with permission from refs. ^59,86^

Researchers have shown interest in using 3D printing technology to create accelerometers. However, currently, only the support structure has been manufactured using 3D printing (Fig. 5a)^59^. Bernasconi et al.^125^ used SU-8 polymer inkjet to print the structure of an inertial accelerometer with a seismic mass and two springs. The accelerometer conforms to high-precision applications, confirming the applicability of IP in inertial MEMS manufacturing. Ge et al. developed a micromachining process combining laser film micromachining and SLA technology, and prepared a piezoelectric MEMS accelerometer based on polyvinyl fluoride (PVDF) films. It is comparable to the most advanced PZT accelerometers in terms of charge sensitivity and noise density, and has a much smaller area than the previous most advanced organic piezoelectric MEMS accelerometers^126^.

MEMS actuators play a critical role in converting electrical signals into mechanical motion, enabling precise control and manipulation at the microscale. The integration of 3D printing technology with MEMS actuators has opened up new possibilities for their design, fabrication, and functionality. Below, we describe some of the published 3D-printed MEMS actuators, which include electronic circuit devices, functional actuators, and some components of miniature systems.

Conductive polymers can be used in the manufacture of microelectronic circuit devices. On a flexible PDMS substrate, some 3D conductive microstructures, including microgrids, micro-snowflakes, micro-springs, micro-honeycomb, and vertical microtube-based arrays (Fig. 5b), were fabricated from OS composite resin (0.5 wt% OS) using the MPL method^86^. This material can also be used to manufacture various electrical components (Fig. 5c) and micro-capacitors (Fig. 5d). The potential of MPL microelectronic device manufacturing process based on OS composite resin is demonstrated.

Polymers can also be used as a sacrifice layer in the manufacturing process, microelectronic circuit devices can be fully 3D printed^68^ or can be combined with other micromachining technologies, only a part of the printing. Chung et al. applied IP technology to print a four-terminal MEMS relay and inverter, which consists of two movable beams placed on the plane, respectively as the body electrode and channel, with good switching control characteristics. The inverter also exhibits abrupt transitions with ultralow static power consumption^127^.

3D printing was utilized for creating support structures in the production of electrothermal actuators^67^, and the choice of printing technology affects the resulting performance. Ulkir manufactured the electrothermal bi-directional actuator using 2PP and DLP respectively, which can shift in both directions. It was found that DLP is more suitable for making asymmetrical structures and has a larger displacement range of 8 μm^89^.

In some miniature systems, 3D printing is mostly used for the preparation of some complex microstructures or package structures. Viviani et al.^128^ created a layer of polymer that reduces vibration by applying an IP technique onto a spring layer of a MEMS device. They examined the interaction between the ink and the substrate, and used a stepped mesh approach to construct a vertical wall structure. The findings indicated that the presence of the polymer damping layer does not have an impact on the mechanical characteristics of the floating spring. However, it does serve to dampen vibrations. Ferrara-Bello et al.^129^ employed 3D printing technology to fabricate the structural component of the microgripper. This technology offers exceptional precision and adaptability, making it well-suited for micromanipulation applications. Alteriis et al.^130^ fabricated redundant inertial measurement units using custom 3D-printed components. Motohashi et al. have created a polyvinyl chloride (PVC) gel micropump that uses a 3D-printed main frame to clamp a PVC gel sheet between three sets of anode and cathode electrodes, after which a voltage is applied to these electrodes in turn to create a peristaltic deformation of the gel sheet, which pushes the liquid and creates a one-way flow^131^. Xie et al. have designed a scissor-structured amplifier made with additive manufacturing technology that converts tiny vibrations into powerful, higher-amplitude vibrations that, coupled with a piezoelectric stretch actuator, could be used in a new type of tactile display for people who are blind or have low vision^132^. Lee et al.^133^ utilized SLA technology and hydrogel material to fabricate a check valve for the treatment of hydrocephalus. This valve effectively reduces backflow leakage and regulates the flow of cerebrospinal fluid. Andres Ferrara-Bello et al.^134^ utilized FDM to fabricate an inexpensive micro-positioning system by employing a piezoelectric buzzer as a piezoelectric brake. The micropositioner has the capability to combine a micro operating system with the microgripper. Soreni-Harari et al.^135^ have presented a technique for fabricating three-dimensional micro-scale multi-material structures using 2PP. Flexible materials and rigid materials can be securely bonded together, with a cambium precision of less than 3 µm. This technique is applicable for the production of miniature robots and MEMS devices. Economidou et al.^136^ fabricated hollow microneedles utilizing SLA technology, which were combined with MEMS to enable a regulated and individualized transdermal medication delivery approach.

Calibration and interaction between 3D-printed devices and MEMS devices can mutually enhance their development, fostering technological advancement. Cayll et al. designed and manufactured a MEMS dynamic mechanical analyzer that can be directly integrated with the 2PP process to evaluate the dynamic mechanical properties and tensile loads of 2PP fabricated nanowires^137^. Garcia et al. proposed a method for calibrating MEMS accelerometers using 3D-printed polyhedra. Up to 14 sensors can be accommodated on each polyhedron, and these sensors can be calibrated simultaneously. This method significantly reduces the root mean square error of sensor output, reduces the cost, and improves the accuracy^138^. Galati et al. combine additive manufacturing technology to propose a new machining method that integrates design, manufacturing, and machining issues, improving mechanical accuracy and component performance^139^. They made guides and mounts to support and move MEMS devices.

### 3D-printed microfluidic devices

The concept of microfluidics was initially propounded by Manz et al.^140^ in 1990. However, the early fabrication processes for microfluidic devices were the process used to fabricate semiconductor devices. The fabrication processes were characterized by complexity and elevated costs, which limited the early development of microfluidic. The constraints surrounding microfluidics underwent a transformative shift with the introduction of soft lithography in 1998^141^. Soft lithography enables the production of microfluidic chips using PDMS, which makes it possible for laboratories to be equipped to produce microfluidic chips. However, this method still required the use of specialized equipment for the production of microfluidic chips and was not widely available. The emergence of 3D printing technology has simplified the manufacturing process of microfluidic devices, allowing the development of microfluidics to break through the bottleneck. The pioneering utilization of 3D printing in microfluidics dates back to 2002 when McDonald et al.^142^ employed Melted Jet Modeling printing technology to print diverse PDMS molds.

**Fig. 6:**
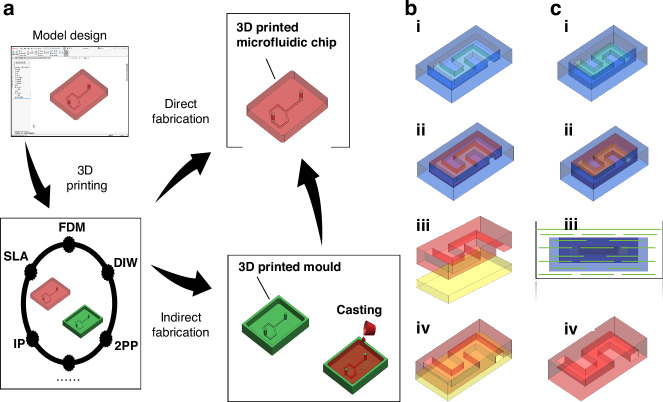
**Manufacturing process for 3D-printed microfluidic devices**. **a** 3D printing microfluidic device manufacturing process. **b** Non-sacrificial mold method. (**i**) Printing the required molds using 3D printing technology. (**ii**) Cast a material such as PDMS into the mold. (**iii**) Remove the resulting cast part from the mold after the cast material has cured and bond it to a PDMS plate or glass substrate to form a closed channel. (**iv**) The final microfluidic device is obtained. **c** Sacrificial mold method. (**i**) Printing the desired mold using 3D printing technology. (**ii**) Cast a material such as PDMS into the mold. (**iii**) After the casting material has solidified, the mold along with the cast parts is immersed in a solution that can dissolve the mold. (**iv**) The final microfluidic device is obtained

Presently, 3D-printed microfluidic devices are being used in a variety of fields. The role of 3D printing technology is to perform direct and indirect manufacturing of microfluidic devices (Fig. 6a). Direct fabrication involves the immediate production of microfluidic devices using 3D printing technology. Conversely, indirect fabrication entails the initial creation of molds through 3D printing technology, followed by the use of these molds to cast microfluidic devices. In this section, we will delve into the materials and manufacturing processes integral to both direct and indirect fabrication of 3D-printed microfluidic devices, outlining their diverse applications across various fields. Ultimately, we will explore the limitations associated with 3D-printed microfluidic devices.

### Direct fabrication of 3D-printed microfluidic devices

The 3D printing technologies commonly employed for direct manufacturing of 3D-printed microfluidic devices include FDM, IP, and VP (SLA and DLP). These 3D printing technologies are all well established and have their own advantages in terms of print results, print resolution, print speed, and material selection.

In the direct fabrication of 3D-printed microfluidic devices, the manufacturing process is carried out based on the respective principles of 3D printing technology. After printing, it is necessary to carry out the corresponding post-processing according to the different characteristics of different printing technologies. The primary distinction among each 3D printing technology lies in the choice of printing materials.

For FDM, the predominant materials encompass various thermoplastic polymers, each offering distinct properties. Among the commonly utilized materials are PLA, polycarbonate, ABS, poly(ethylene terephthalate) (PET), and poly(ε-caprolactone) (PCL), with the PCL being recognized as a biocompatible option^143^. Additionally, materials such as polystyrene (PS), polymethylmethacrylate, polyether ether ketone, polyamide, or nylon find application in this technology.

In comparison, IP, while offering advantages over FDM^144^, has a more restricted range of compatible materials. These include light-curing resins/photopolymers, methacrylates, liquid suspensions, elastomers, and ceramic-based inks.

Vat Polymerization, which includes SLA and DLP, shares similarities with IP but exhibits a broader spectrum of materials. This encompasses light-curing resins/photopolymers, (meth)acrylate-based formulations, epoxy resins, virgin acrylic resins, elastomers, ceramic-based formulations, as well as composites and hybrid photopolymers, thereby expanding the range of materials beyond those applicable in IP.

Owing to its straightforward process flow, high flexibility, and cost-effectiveness, direct manufacturing is extensively adopted for the validation fabrication of 3D-printed microfluidic devices in laboratory settings. This approach has found widespread use across diverse fields, showcasing numerous innovative applications.

### Indirect fabrication of 3D-printed microfluidic devices

The distinction between indirect fabrication and direct fabrication of 3D-printed microfluidic devices primarily lies in the sequence of steps, wherein indirect fabrication entails printing the mold first and subsequently completing the microfluidic device through casting. Various 3D printing technologies are employed for the indirect manufacturing of molds, with common techniques including FDM, SLA, and IP.

These 3D printing techniques, based on the manufacturing process and materials used, can be further categorized into non-sacrificial and sacrificial mold printing methods for microfluidic device fabrication. Non-sacrificial mold printing involves the 3D printing of the mold for the microfluidic device. The printing material chosen should not react with the casting material. Following the printing of the mold, the material for the microfluidic device is cast into it. It is noteworthy that microfluidic devices obtained through casting with non-sacrificial mold printing methods typically have unsealed microfluidic channels. Consequently, it is necessary to wait for the material to cure, then remove the microfluidic device from the mold, and subsequently bond it to the other half of the device or to a glass base^145^. This completes the manufacturing process of microfluidic devices (Fig. 6b). In the sacrificial mold process, the initial step involves 3D printing the mold for the microfluidic device using a material that is easily dissolvable. Subsequently, the material for the microfluidic device is cast into the mold. After curing, the mold is dissolved using a substance that reacts with it, and the resulting device is cleaned with a suitable agent, such as water, to finalize the microfluidic device^146,147^ (Fig. 6c).

Fused deposition modeling (FDM) is versatile in its application for both sacrificial and non-sacrificial molds in the indirect manufacturing of microfluidic devices. When performing indirect manufacturing of microfluidic devices for sacrificial molds, FDM typically prints the molds using two materials, polyvinyl alcohol (PVA), which is soluble in water, and ABS, which is soluble in acetone^146,147^. Conversely, in the indirect fabrication of microfluidic devices with non-sacrificial molds, common materials such as PLA and ABS are typically employed for mold printing^145^. These materials are chosen for their compatibility with the casting material and the overall manufacturing process.

Stereolithography (SLA) technology has gained widespread adoption for the fabrication of non-sacrificial soft lithography molds utilized in PDMS microfluidic devices. Its principle is similar to that of soft lithography and the fabrication steps have been simplified, making it more popular for the fabrication of microfluidic devices. The printing materials used in SLA are mainly resin materials. It essential to note that some resin materials may react with microfluidic device materials such as PDMS or even inhibit the curing of microfluidic devices. As a result, the SLA method for mold printing often necessitates distinct post-processing operations, contingent upon the specific combination of materials chosen for printing the mold and casting the microfluidic device^148^. These considerations are crucial to ensure compatibility and optimal performance in the fabrication process.

IP methods, including Polyjet and Multijet, are versatile and can be applied to both sacrificial and non-sacrificial mold printing processes. Given that IP involves the use of both print and support materials, it is crucial to ensure the complete removal of the support material without leaving any residues after the mold has been printed. In the case of Polyjet printing, the support material typically comprises a combination of acrylic monomer, polyethylene, propylene, and glycerin. High-pressure water jetting is commonly employed to remove this support material effectively. On the other hand, for Multijet printing, the support material is typically wax, which can be melted away in water.

Compared to direct manufacturing, the indirect fabrication of 3D-printed microfluidic devices possesses the advantage of efficiently and cost-effectively producing these devices in large quantities. Furthermore, this process aligns seamlessly with traditional microfluidic fabrication materials, notably the widely utilized PDMS. Consequently, researchers across diverse fields are currently exploring methodologies to attain a considerable number of low-cost and high-precision 3D-printed microfluidic devices through indirect manufacturing

#### Applications of 3D-printed microfluidic devices

Currently, the applications of 3D-printed microfluidic devices are focused on the fields of biology, chemistry, and medicine. The main fields of application are biology and chemistry, while other applications in medicine and other fields are based on research in biology and chemistry. Most of the applications in the biological field are at the level of cells, tissues, and microorganisms. Applications in the field of chemistry are chemical sensors and wearable sensors for monitoring human body signs.

**Fig. 7:**
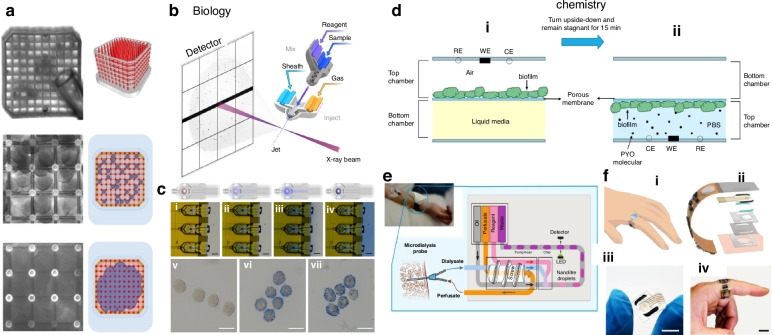
**3D-printed microfluidic devices in biology and chemistry**. **a** Microvascular network and tissue generation process^149^. The top row shows a schematic diagram in which the spheres and generated tissues are shown in blue, and the bottom row shows the experimental tissue generation process. The right image of the bottom row is an image of the spheres fused into solid tissue after 24–36 h of incubation on the chip, scale bar 250 µm. **b** A miniature three-dimensional mixer with greatly improved mixing efficiency for mixing-injection of time-resolved SFX^150^. Protein crystal suspensions can be injected into the X-ray interaction region for diffraction imaging. **c** Perfusion microfluidic device is used to treat osmotically stabilized HEK293 cell spheroids with trypan blue, scale bar 200 µm. (**i**)–(**iv**) staining and washing process; (**v**) untreated control spheres; (**vi**) spheres stained on the bench under standard static conditions; (**vii**) spheres stained by the delivery of trypan blue stain through the fluidic device as shown in (**i**–**iv**)^151^. **d** Novel dual-chamber microfluidic device for real-time in situ electrochemical monitoring of gas-liquid interfaces^152^. (**i**) Detection of biofilm formation on the ALI using a three-electrode integrated dual-chamber microfluidic device. (**ii**) PYO graphical representation of biofilm detection on ALI using a three-electrode integrated dual-chamber microfluidic device. **e** Schematic diagram of device operation^153^. Dialysate is delivered from the pump to the microfluidic chip. Reagents and analytes react in the chip to produce a measurable optical response. The image in the upper left corner illustrates the sensors used to monitor tissue in a clinical setting. **f** Schematic of a flexible wireless microfluidic wearable patch^154^. (**i**)–(**ii**) Schematic diagrams. (**iii**) Picture of the wearable patch. (**iv**) Photograph of a fully integrated wireless wearable patch worn on the finger. Scale bar is 1 cm. Reprinted with permission from refs. ^149–154^

3D-printed microfluidic devices are the most widely used in the field of biology. A large number of teams are working on the application and development of microfluidics in various branches of biology. The branch directions of biology can be categorized into developmental biology, biochemistry, cell biology, biomarkers, biophysics, microbiology, and biotechnology. Grebenyuk et al.^149^ present a microvascular network 3D printed by 2PP technology (Fig. 7a). This microvascular network allows for large-scale perfusion of tissues and can be used for tissue culture. And its ability to solve major problems of oxygen, nutrients and growth factors, and small molecule supply as well as waste removal has important implications in developmental biology. And in the field of biochemistry, Knoška et al.^150^ customized microfluidic nozzles and mixers for time-resolved structural biology of X-ray free electron lasers (XFELs) through the 2PP technique (Fig. 7b). This enables aberration-free imaging of 3D fluid dynamics during manipulation, making it possible to decipher the mechanisms and regulation of dynamic biological processes. McLennan et al.^151^ printed a microfluidic chip using 2PP printing technique (Fig. 7c). The device is capable of promoting cell growth and morphological changes and can be used for the delivery of media, dyes, and biomolecules to fulfill the functions of cell growth, staining, and cell phenotypic changes, respectively. This makes it possible to realize the key technology of in vitro cell culture in cell biology.

In the field of chemistry, 3D-printed microfluidic devices can be used for the detection of various electrochemical signals. Zhang et al.^152^ present a novel two-compartment microfluidic device using a real-time in situ electrochemical monitoring of Pseudomonas aeruginosa biofilms grown on a gas-liquid interface and their antibiotic susceptibility (Fig. 7d). PDMS films for detection are obtained by indirect fabrication using stereolithography 3D printing. The device enables real-time, non-invasive monitoring of biofilm growth for chlorophyll (PYO), enabling rapid, low-cost ALI biofilm studies and antimicrobial testing. Nightingale et al.^153^ develop a 3D-printed microfluidic wearable droplet sensor for monitoring the concentration of biomolecules in tissues (Fig. 7e). A PDMS microfluidic chip cast using a 3D printing mold is combined with a peristaltic pump to form a wearable droplet sensor for monitoring biomolecule levels. Non-invasive 3D-printed microfluidic sensors have also been developed in recent studies. Ye et al.^154^ develop a wearable aptamer nano biosensor for non-invasive female hormone monitoring (Fig. 7f). The sensor utilizes IP technology for continuous printing to obtain the sensor patch. Non-invasive monitoring of estradiol (female hormone) can be achieved through sweat analysis, opening up the potential of wearable sensors for non-invasive, personalized reproductive hormone monitoring.

3D-printed microfluidic devices have other innovative engineering applications. Li et al.^155^ proposed a fish-like antifouling membrane bionic filtration device. Fish gill structure is mimicked by 3D printing gill raker-like structures directly on the membrane surface to achieve anti-fouling and anti-clogging functions for wastewater filtration. Dai et al.^156^ propose a bionic juxtaposed compound eye. Insect-like bionic compound eyes are obtained by casting using 3D printing indirect manufacturing, which in conjunction with commercial imaging sensors enable the capture of full-color wide-angle panoramic views and precise positional tracking of point sources. It will be used in a variety of applications such as photonics, sensing, and imaging.

### Shortcomings of 3D-printed microfluidic devices

The size of the microchannels in a microfluidic device is the key parameter, and although most current studies claim to be investigating 3D-printed microfluidic devices, the microchannels obtained from most commercial 3D printers are usually larger than 100 µm when actually printed. Of course, there are also teams of academics working on how to realize 3D printing of microchannels smaller than 100 µm. Gong et al.^157^ achieved the printing of microfluidic channels with cross-sectional areas as small as 18 μm × 20 μm using a customized 3D printer. Vedhanayagam et al.^158^ have achieved low-cost, fast 3D printing of soft lithography molds that can be used to fabricate microfluidic channels of less than 100 µm with a commercial high-resolution digital light projection (DLP) resin printer.

The transparency of current 3D-printed microfluidic devices is deficient, and when printing with FDM, air bubbles are trapped within the layers, which is a major reason why the devices become opaque. In addition, 3D printers often use rough build surfaces to improve the adhesion of the first layer, which reduces the transparency of the resulting device and requires post-processing such as sanding and polishing (i.e. immersing into ethanol) to improve transparency. Bucciarelli et al.^159^ optimized the process of DLP printing by DoE strategy and demonstrated that the optimized method is effective in enhancing the transparency of microfluidic chips by developing a DLP printing process to produce chips for biomedical applications.

Since microfluidic devices are well suited for biological applications, the biocompatibility of the materials used in the devices is a key parameter for the biological applications of 3D-printed microfluidic devices. Studies have found that FDM materials such as PLA and ABS are biocompatible and have not produced any toxic effects^143^. However, SLA and inkjet resins are normally non-biocompatible, even PDMS devices produced using 3D printing molds showed toxic leachate.

Surface roughness is an unavoidable problem with 3D printing layer-by-layer manufacturing methods, with FDM having the highest surface roughness^160^. Higher surface roughness affects the transparency of directly fabricated 3D-printed microfluidic devices and the smoothness of their microchannels, while in indirect fabrication it affects the adhesion of the microfluidic devices to glass coverslips or PDMS blocks. However, the most common method to deal with the surface roughness of 3D-printed microfluidic devices is to cover the surface of the microchannel with a layer of PDMS or other materials to make it smooth^153^.

## Outlook and future directions

In the last twenty years, there has been notable advancement in the development of micro and nanodevices through the use of 3D printing technology. This paper covers several aspects of 3D printing technology such as the characteristics, materials, and applications, based on available literature. High-resolution 3D printing technologies, such as 2PP, DIW, and EHDP, have proven to be useful in creating microelectronic components and microfluidic devices with submicron features. Furthermore, the techniques of SLA, FDM, and other emerging 3D printing technologies have reached a higher level of maturity in the fabrication of micro-devices.

The primary use of 3D printing in sensor manufacture is for creating the auxiliary and support structures of the sensor. The utilization of 3D printing technology to create microchannels with high aspect ratios is a novel approach to address the needs of gas or liquid sensors. Several sensors incorporate electrodes, dielectric layers, and specialized thin films with specific topologies, which play a crucial role in enabling sensor functionality. The ideal solution is in the utilization of versatile, high-resolution 3D printing techniques that have the ability to create structures with a high aspect ratio. Furthermore, functional polymer materials and ink materials have achieved a state of full development and can be utilized for the manufacturing of 3D-printed functional components. This strategy facilitates the integration of multiple components or functionalities into a single printing device, thereby reducing the need for assembly and enhancing the overall performance of the device. Currently, there is a scarcity of study in this field. However, the functional composition of sensors, such as piezoelectric structures created through 3D printing, will increasingly emerge as a significant area of advancement in the future.

When comparing 3D printing to traditional 2D planar micromachining technologies like soft lithography, it becomes evident that 3D printing offers several advantages such as shorter production cycles and less material waste in constructing intricate three-dimensional structures. This is especially significant for MEMS devices that require complicated three-dimensional structures. Conventionally, the expense of producing an individual device often diminishes as the quantity of devices needed escalates. By incorporating 3D printing technology in the manufacturing process of MEMS devices, there is a notable reduction in expenses, resulting in a cost advantage for producing MEMS devices in limited quantities. 3D printing employs additive manufacturing techniques to produce MEMS devices layer-by-layer, reducing the requirement for conventional manufacturing methods and facilitating the incorporation of intricate designs and functions. 3D printing enables the quick production of prototypes and iterative design processes, facilitating the construction and enhancement of MEMS devices. The 3D printing technology provides greater flexibility and feasibility in microgeometric design and manufacturing compared to subtractive processes like machining and laser cutting. This approach enables meticulous manipulation of the design, materials, and performance of the device at the micron scale. 3D printing enables the swift fabrication of intricate structures and facilitates the development of functional modules with novel materials. Nonetheless, the preparation of micro-executive modules cannot be accomplished autonomously. MEMS devices, particularly actuators, necessitate stringent mechanical property standards for materials. Most contemporary reports on MEMS devices are predominantly based on silicon due to its mechanical qualities^161^. The mechanical properties of 3D printing materials are typically challenging to regulate, necessitating the integration of 3D printing with materials produced through other technologies to enhance their mechanical characteristics^3^. Stiffness, strength, and toughness can also be improved by changing the interlayer structure of the material^162^. Nevertheless, the precision of these methods fails to satisfy the demands of microdevice fabrication. Owing to the elevated roughness and diminished tensile strength of 3D-printed structures, 3D printing is predominantly employed in MEMS actuators for the fabrication of support structures, frame structures, and device packages that do not necessitate adjustable mechanical qualities. 3D printing frequently necessitates integration with additional micromachining techniques to create an active mechanical structure that fulfills the specifications, and entirely 3D-printed MEMS actuators have not yet been realized. Various 3D printing technologies employed to fabricate the same device will exhibit distinct mechanical features; for instance, DLP is more adept than 2PP for producing asymmetric actuating structures, enabling a broader displacement range. The selection of 3D printing technology for certain structural features is a prevailing research trend among scholars.

In the preparation process of microfluidic systems, either directly or indirectly, 3D printing has made it possible to create microfluidic channels with high aspect ratios. However, the resolution, smoothness, and transparency of the microchannels created through 3D printing have frequently constrained the progress of microfluidic devices. Additional investigation is required on biocompatible and non-toxic substances, as well as the corresponding techniques for high-precision 3D printing.

The future directions of 3D printing technology involve enhancing printing speed, minimizing expenses, broadening the selection of materials, and accomplishing more intricate structural and functional integration. There is a strong need for small, inexpensive, and accurate micro and nanodevices in various fields such as sensors, electrical circuits, actuators, and microfluidic systems. In order to fulfill these requirements, it is imperative for 3D printing technology to progress further and improve its compatibility with micro and nanodevices. This will establish a strong basis for the future growth and utilization of micro and nanotechnology.
